# Molecular mechanisms regulating cGAS/STING activation in health and disease

**DOI:** 10.1172/JCI204548

**Published:** 2026-07-01

**Authors:** Min-Guk Cho, Rachel Lee, Jaycee Johnson, Gaorav P. Gupta

**Affiliations:** 1 Lineberger Comprehensive Cancer Center,; 2 Curriculum in Genetics and Molecular Biology,; 3 Department of Biochemistry and Biophysics, and; 4 Department of Radiation Oncology, University of North Carolina at Chapel Hill, Chapel Hill, North Carolina, USA.

## Abstract

The cGAS/STING pathway enables cells to sense cytosolic DNA and mount rapid innate immune responses to infection, cellular stress, and tissue damage. While essential for host defense and immune surveillance, inappropriate or sustained activation of this pathway can drive chronic inflammation, autoimmunity, and disease-associated immune dysfunction, which can promote cancer growth. Effective immunity therefore depends on precise regulatory control that restrains cGAS/STING activity under homeostatic conditions while preserving the capacity for swift and robust responses to diverse danger signals. In this Review, we synthesize emerging principles that regulate cGAS/STING signaling across cellular contexts to control signal initiation, amplification, and termination. We discuss how disruption, persistence, or pathological rewiring of these regulatory processes contributes to immune imbalance across health and disease, promoting chronic inflammation, immunosuppression, and tissue pathology, with particular relevance to tumor progression and therapeutic resistance. Finally, we consider how restoring appropriate cGAS/STING regulation, rather than simply enhancing or inhibiting pathway activity, may reestablish immune homeostasis and improve therapeutic outcomes in cancer and other inflammatory diseases, framing the pathway as a dynamic regulatory circuit rather than a simple linear signaling cascade.

## Introduction

Cells continuously monitor their internal and external environments to detect signals of infection, stress, or damage. In particular, nucleic acids that are aberrant in structure or mislocalized act as potent danger signals indicative of microbial invasion or loss of cellular integrity (1–5). Among the pathways that surveil for such threats, the cyclic GMP-AMP synthase (cGAS)/stimulator of interferon genes (STING) axis has emerged as a central mechanism that detects dsDNA in the cytosol and initiates innate immune activation (6, 7). Although initially characterized in the context of defense against DNA viruses, this pathway is now understood to govern immune homeostasis across a wide spectrum of biological contexts, ranging from autoinflammatory syndromes to cancer (1, 8, 9).

Canonical activation of the pathway begins when cGAS binds to dsDNA in a sequence-independent manner (6, 10). This binding induces a conformational change in cGAS, activating its catalytic site to synthesize the second messenger 2′3′-cyclic GMP-AMP (2′3′-cGAMP) from ATP and GTP (6, 11, 12). The generated 2′3′-cGAMP binds to the ER-resident adaptor STING, inducing its oligomerization and translocation to the Golgi apparatus (13). At the Golgi, STING recruits TANK-binding kinase 1 (TBK1), which phosphorylates interferon regulatory factor 3 (IRF3), leading to the transcriptional induction of type I IFNs and inflammatory cytokines (1, 7, 14).

Given the potency of this signaling, cells employ multilayered regulatory mechanisms to prevent inappropriate activation by self-DNA. Under homeostatic conditions, cGAS activity is tightly constrained; for instance, cGAS is tethered to nucleosomes in the nucleus to prevent activation by genomic DNA (15–18), and the nuclear envelope acts as a physical barrier (1). However, when these safeguards fail — due to defects in nucleases (e.g., TREX1), mitochondrial damage, or genomic instability — sustained cGAS/STING signaling can drive pathology (19–22). Notably, gain-of-function mutations in *TMEM173* (encoding STING) cause STING-associated vasculopathy with onset in infancy (SAVI), a severe autoinflammatory disease (23). Similarly, aberrant sensing of self-DNA from damaged mitochondria or micronuclei has been implicated in neurodegenerative conditions such as Parkinson’s disease and cellular senescence (21).

While essential for eliminating threats, the relevance of this pathway is particularly complex in cancer biology. Tumor cells are characterized by oncogene activation, oxidative stress, and impaired DNA repair, which collectively elevate the burden of cytosolic dsDNA arising from micronuclei and chromatin fragments (1, 24). This accumulation can trigger cGAS/STING-mediated antitumor immunity, recruiting cytotoxic T cells to the tumor microenvironment (25). However, many tumors evolve strategies to evade this surveillance, such as epigenetic silencing of cGAS/STING or upregulating ENPP1 to degrade extracellular cGAMP (26–31). Paradoxically, chronic low-level activation of STING in established tumors can shift signaling away from cytotoxic IFN responses toward NF-κB–driven chronic inflammation that supports metastasis and tissue remodeling (22).

Recent studies increasingly position the cGAS/STING pathway not merely as a DNA sensor, but as a multifunctional regulatory node integrating chromatin architecture, metabolism, and immune signaling (10, 13, 16, 17). In this Review, we synthesize the molecular mechanisms governing cGAS/STING activation and regulation. We discuss how disruption or co-option of these processes drives diverse diseases, including autoimmunity, neurodegeneration, and particularly cancer. Finally, we highlight therapeutic strategies to restore immune balance by targeting specific regulatory nodes of this dynamic circuit.

## Origins and restriction of immunostimulatory DNA

Immunostimulatory DNA can stem from both exogenous introduction of foreign nucleic acids and endogenous DNA damage, metabolic stress, transcriptional dysregulation, and genomic instability. Aberrant localization of dsDNA in the cytosol serves as a damage-associated molecular pattern (DAMP) that stimulates innate immune signaling. Endogenous DAMPs contribute to proinflammatory responses, and dsDNA-derived DAMPs result in cGAS activation. Regulation of cGAS-mediated immune responses through genome maintenance and restriction of cytosolic DNA is essential to prevent chronic inflammatory conditions.

**Figure 1:**
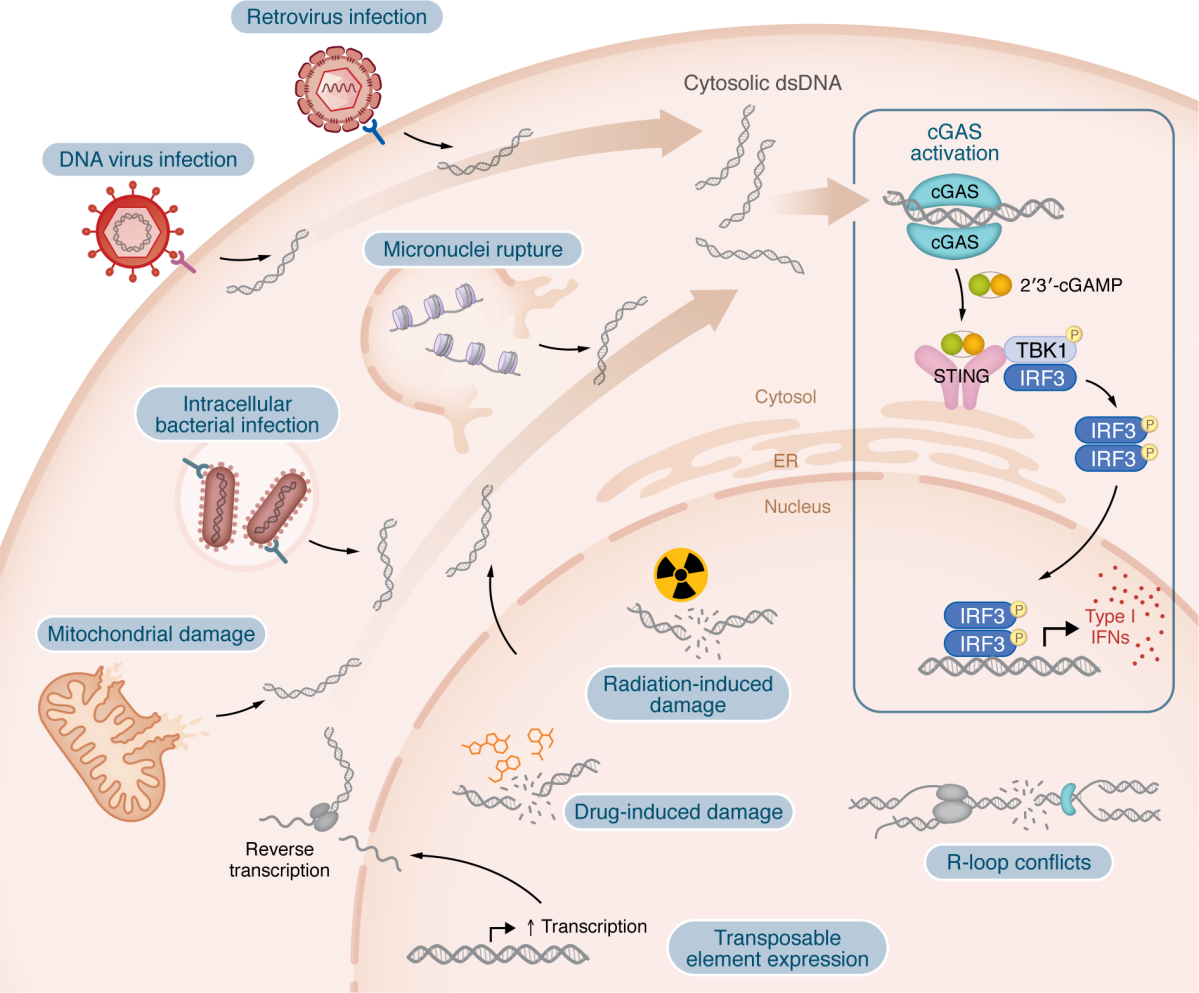
Sources of immunostimulatory DNA. Cytosolic dsDNA fragments serve as a danger signal, and their recognition by cGAS elicits inflammatory signaling responses. Exogenous sources of immunostimulatory dsDNA are primarily microbial, including DNA viruses, retroviruses, and intracellular bacteria. Endogenous sources of immunostimulatory dsDNA arise from DNA damage, metabolic stress, transcriptional dysregulation, and genomic instability. DNA damage is caused by irradiation, anticancer drugs, and transcription replication conflicts, which can generate cytosolic dsDNA fragments. Metabolic stress can disrupt mitochondrial membranes and result in mtDNA release. Aberrant chromosome segregation can lead to formation of micronuclei whose disordered structure leaves them prone to membrane collapse, thereby exposing micronuclear dsDNA to cytosolic cGAS. Derepression of transposable elements can lead to accumulation of cytosolic cDNA and RNA-DNA hybrids. Upon binding these cytosolic dsDNA species, cGAS synthesizes 2′3′-cGAMP, which then activates STING to mediate a signaling cascade that leads to expression of type I IFN.

Effective detection of microbes is critical for induction of innate immune signaling and clearance of infections. cGAS was first described in the context of antiviral innate immunity and is activated by DNA viruses, retroviruses, and intracellular bacterial DNA (6, 32, 33) (Figure 1). Initial studies performed in cGAS*-*deficient mice demonstrated a lack of type I IFN response and subsequent mortality upon challenge with the DNA virus HSV-1 (32). More recently, the spatial context of cGAS activity in sensing viral DNA has been expanded to include the nuclear soluble fraction of cells (34).

cGAS is also activated by intracellular DNA sources to stimulate sterile inflammation. Micronuclei are membrane-bound organelles that can arise during mitosis in response to mis-segregated chromosomes or chromosomal fragments (35). The intrinsic instability of these structures due to lamina disorganization leaves micronuclei susceptible to collapse (35). Membrane disruption of micronuclei results in cytosolic exposure of DNA fragments, which can lead to rapid accumulation of cGAS in cycling cells, and progression through mitosis serves as a critical factor for cGAS localization to micronuclei (1, 36). Further, prolonged cell cycle arrest has been shown to inhibit cGAS-mediated signaling in response to micronuclei (37). Notably, recent reports challenge this paradigm, suggesting that while cGAS localizes to micronuclei, activation following DNA damage is rare. They suggest that chromatin bridges rather than micronuclei may serve as dominant triggers and that micronuclei induced by radiation, replication stress, or chromosome segregation errors often fail to elicit productive cGAS/STING signaling (38–40).

Autophagy is a homeostatic process that restricts excessive or prolonged inflammation. cGAS activation has been implicated as a trigger of autophagy induction to inhibit proliferation of cells in replicative crisis (41). Additionally, cGAS has been shown to interact with autophagy machinery to direct clearance of micronuclei and suppress innate immune signaling (42).

Oxidative stress and metabolic dysfunction are associated with mitochondrial damage that can drive pathologic inflammation and aging. In these sterile contexts, DAMPs such as cytosolic mtDNA can act as endogenous triggers of cGAS/STING signaling (Figure 1). Deficiency in the mtDNA-binding protein TFAM drives mtDNA instability and aberrant nucleoid packaging, leading to mtDNA leakage into the cytosol where the fragments are bound by cGAS (21). Similarly, recent work has demonstrated that voltage-dependent anion channel 2 (VDAC2) is a mediator of mtDNA-induced cGAS signaling, such that loss of VDAC2 leads to unrestrained BAK activation and subsequent mitochondrial damage (43). Mitochondrial stress can also be a by-product of radiotherapy, resulting in mtDNA release and cGAS activation, and, importantly, may serve as a critical damage signal to induce systemic inflammatory responses through exosome and cell-free DNA trafficking (44). mtDNA has recently been implicated in a negative feedback loop in which release of mtDNA activates the N-degron pathway to direct cytosolic DNA to lysosomes for degradation to prevent excessive cGAS-mediated inflammation (45).

Transposable elements (TEs) are highly abundant and repetitive sequences comprising more than 50% of the human genome (46). TEs are canonically silenced through epigenetic modification to restrict genome instability and resulting inflammatory responses, but TEs are commonly derepressed under stress conditions, including cancer and aging (47, 48). TEs have also been implicated as triggers of cGAS activation, as reverse transcription of these elements in the cytosol exposes cDNA and RNA-DNA hybrids to cGAS sensing (Figure 1) (49–51). DNASE2 serves as a regulator of cytoplasmic DNA, including TE DNA, but it is downregulated in senescent cells, promoting senescence-associated inflammation driven by cGAS signaling (52). This was demonstrated in a study in which derepression of LINE-1 in aged tissues led to accumulation of LINE-1 cDNA in the cytosol and subsequent cGAS-mediated sterile inflammation, which could be abrogated with reverse transcriptase inhibitors (49, 50).

**Figure 2:**
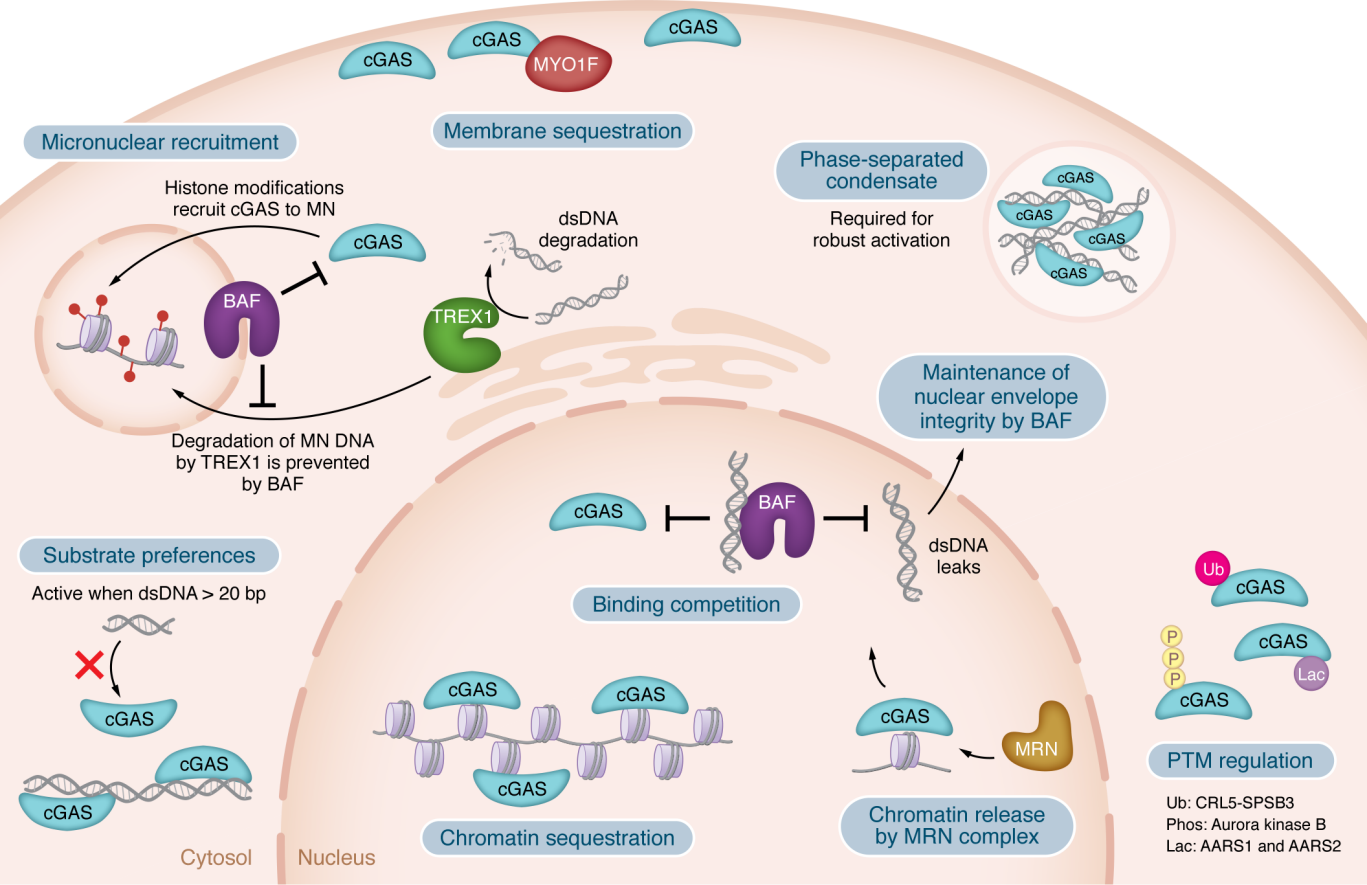
Licensing cGAS: chromatin gating, nuclear envelope integrity, and spatial control. To enable proper response of the innate immune system, cGAS must be tightly regulated and only activated at specific moments. Inside the nucleus, cGAS is sequestered on chromatin in an inactive state. Under specific conditions, the Mre11-Rad50-Nbs1 complex releases chromatin-bound cGAS, allowing its translocation and sensing of cytosolic dsDNA. cGAS activation is enhanced upon binding to dsDNA and forming phase-separated condensates, which create an environment that promotes efficient and robust enzymatic activity. cGAS activity is also regulated by posttranslational modifications (PTMs), including ubiquitination, phosphorylation, and lactylation, which influence cGAS stability, localization, and activation. In micronuclei (MN), histone modifications regulate the recruitment and localization of cGAS to micronuclear dsDNA. BAF plays multiple roles in cGAS regulation. BAF modulates cGAS access to micronuclear dsDNA primarily by maintaining nuclear and micronuclear envelope integrity, thereby limiting exposure of self-dsDNA to the cytosol. In addition, BAF blocks TREX1 activity in micronuclei to prevent degradation of dsDNA. cGAS localization is further regulated through trafficking to distinct cellular compartments, including recruitment to the plasma membrane by MYO1F. cGAS preferentially binds and responds to dsDNA longer than 20 bp. Together, these mechanisms regulate cGAS activation and the subsequent induction of STING-dependent inflammatory signaling pathways.

Replication stress causes DNA double-strand breaks, in part due to RNA-DNA hybrid accumulation in the form of R-loops and conflicts between transcription and replication complexes (Figure 1) (53). Damage induced by R-loop accumulation was suggested as a source of genome-derived cytosolic dsDNA in lymphoma models, where high levels of cytosolic DNA were correlated with a type I IFN response, and enzymatic resolution of R-loops decreased cytosolic DNA burden and inflammatory signaling (54). Cytosolic RNA-DNA hybrids derived from failed R-loop processing have since been shown to serve as direct immunostimulatory substrates of cGAS (55). Nucleases play a pivotal role in R-loop resolution and restriction of DNA fragments that escape the nucleus. RNASEH1 functions in nuclei and mitochondria to resolve R-loops through cleavage of the RNA strand (54). Similarly, RNASEH2 is recruited to sites of active transcription to resolve cotranscriptional R-loops, and deficiency in RNASEH2 is associated with micronuclei formation and cGAS activation (56). SAMHD1 is a dNTPase that serves as a key regulator of replication stress, acting through stimulation of MRE11 to prevent release of immunostimulatory DNA (57). TREX1 is a 3′→5′ exonuclease that restricts accumulation of cytosolic DNA (Figure 2) (58).

Inherited mutations in RNA-DNA hybrid processing pathways, including mutations in RNASEH2 subunits and *TREX1*, result in severe cGAS-dependent type I interferonopathies, including Aicardi-Goutières syndrome, and have also been associated with autoimmune conditions (20, 56). The tumor suppressor p53 has been shown to mediate degradation of TREX1 to activate cGAS and prevent oncogenic transformation (59). TREX1 can also be targeted for degradation by a SPOP-associated ubiquitin ligase but is conversely stabilized by the deubiquitinase USP7, wherein loss of SPOP function or overexpression of USP7 in cancers is associated with increased TREX1-mediated restriction of cytosolic dsDNA and poor response to immunotherapies (60). Enzymes regulating the architecture of DNA during transcription, including helicases and topoisomerases, also play a vital role in regulating R-loops to prevent fork conflict–induced DNA damage and cytosolic release. For example, the helicase SETX contributes to resolving double-strand breaks through Rad51 recruitment by unwinding the RNA-DNA hybrid component of R-loops flanking break sites (61). SETX has also been established as a critical regulator of MYC-induced replication stress, with loss of SETX exacerbating genome instability (62). Additionally, helicase DXH9 interacts with R-loops, and its depletion increases DNA damage markers (63). Topoisomerase I prevents R-loop formation by limiting torsional stress and preventing reannealing of nascent RNA (54, 64, 65).

Cancer treatments are a major source of stress signals that are a critical mediator of therapeutic response (Figure 1). Intercalating agents distort DNA structures, leading to strand breaks and escape of DNA fragments into the cytosol, where cGAS is engaged (66). Similarly, induction of cGAS/STING has been observed upon treatment with several anticancer drug classes including platinum-based agents, microtubule-targeting agents, and topoisomerase inhibitors such as etoposide (67–69). Therapies targeting DNA damage response pathways, such as PARP inhibition, have also been shown to induce cell-intrinsic cGAS signaling (70). Radiotherapy is commonly employed to induce DNA damage for tumor control, which has been shown to induce cGAS-mediated innate immune activation, and this effect is further potentiated by combination with CHEK1/2 inhibition to promote cell cycle progression, resulting in increased micronuclei formation (71).

Taken together, these findings indicate that a fine balance must be maintained between danger signals that activate cGAS-mediated innate immunity and restriction of immunostimulatory nucleic acids to prevent pathologic inflammation.

## cGAS licensing and regulation

Given the abundance of genomic DNA within cells, it is perhaps not surprising that there are multiple licensing mechanisms that regulate cGAS to prevent overactivation. For example, cGAS activation requires its ability to bind dsDNA and form a 2:2 cGAS/DNA oligomer, which coalesces to form a phase-separated condensate in which 2′3′-cGAMP can be produced (72–74). This biophysical property is governed by the disordered N-terminal domain of cGAS and blocks interactions with other protein regulators that may prevent cGAS from achieving its enzymatic function (Figure 2) (18, 19, 73, 74).

In addition, cGAS is prevented from recognizing self-DNA in part through its sequestration to nuclear chromatin (16, 75). Cryo-electron microscopy structures of cGAS show its binding to the acidic patch of nucleosomes in a conformation that occludes the DNA binding pocket on cGAS that is required for its cGAMP-producing enzymatic activity (15–17, 75, 76). This is moderated by two arginines that interact with acidic residues in histone H2A (15–17, 75, 76). It is possible that certain histone modifications may disrupt or enhance cGAS binding to nucleosomes (77, 78). Additionally, under certain conditions like DNA damage, cGAS can be released from the nucleosome by the DNA double-strand break–sensing Mre11-Rad50-Nbs1 complex (79) (Figure 2). This repositions cGAS and allows it to sense DNA fragments in cytosolic compartments and generate cGAMP to trigger STING-dependent inflammation (79).

cGAS activation is also limited by factors including localization, DNA fragment length, and competition with other dsDNA binders (Figure 2). As noted above, the unstructured N-terminal tail of cGAS determines its interactions with other proteins and in turn plays a role in its cellular and chromatin localization (80). cGAS also contains a nuclear export signal (81). Thus, cGAS can be found in most areas of the cell and serves as a surveillance enzyme for any signs of pathogen or host-derived cytosolic DNA. Interestingly, longer dsDNA fragments are more likely to elicit cGAS activation than shorter lengths (82). Additionally, in cases where cGAS is free and can bind to self-DNA, it can be outcompeted by other dsDNA-binding proteins, dampening the innate immune response. During nuclear envelope rupture, as occurs during mitosis, free cGAS gathers at the nuclear periphery, where it is outcompeted for dsDNA binding by Barrier to autointegration factor (BAF), thereby restricting its activation (Figure 2) (18). BAF plays a multifaceted role in cGAS regulation through maintenance of nuclear envelope integrity and prevention of DNA release from micronuclei, and it has also been found to block the exonuclease TREX1 from accessing micronuclear DNA, preventing degradation of DNA, which may stimulate cGAS activity (83–85). The methyl-CpG-binding protein 2 (MeCP2) is also known to outcompete cGAS for DNA binding and dampen the innate immune system response (86).

Finally, cGAS is regulated by posttranslational modifications (PTMs) and through trafficking by other proteins. cGAS can be lactylated, phosphorylated, acetylated, and ubiquitinated by a variety of enzymes. AARS1- or AARS2-mediated lactylation of cGAS in its N-terminus and Aurora kinase B–mediated hyperphosphorylation of the same domain both prevent cGAS phase separation and limit DNA binding (Figure 2) (87, 88). A member of the Cullin-ring family of ubiquitin ligases, CRL5-SPSB3, can ubiquitinate cGAS to degrade it as a form of cell cycle control (89). Acetylation of cGAS’s N-terminal region has also been shown to decrease activity and DNA binding (90). Additionally, cGAS is known to interact with other protein complexes that regulate its function. One such protein is MYO1F, which facilitates cGAS localization to the cellular membrane (Figure 2) (91). This shuttling of cGAS to different locations within the cell helps prevent its interactions with self-DNA.

## STING signal initiation, propagation, and resolution

**Figure 3:**
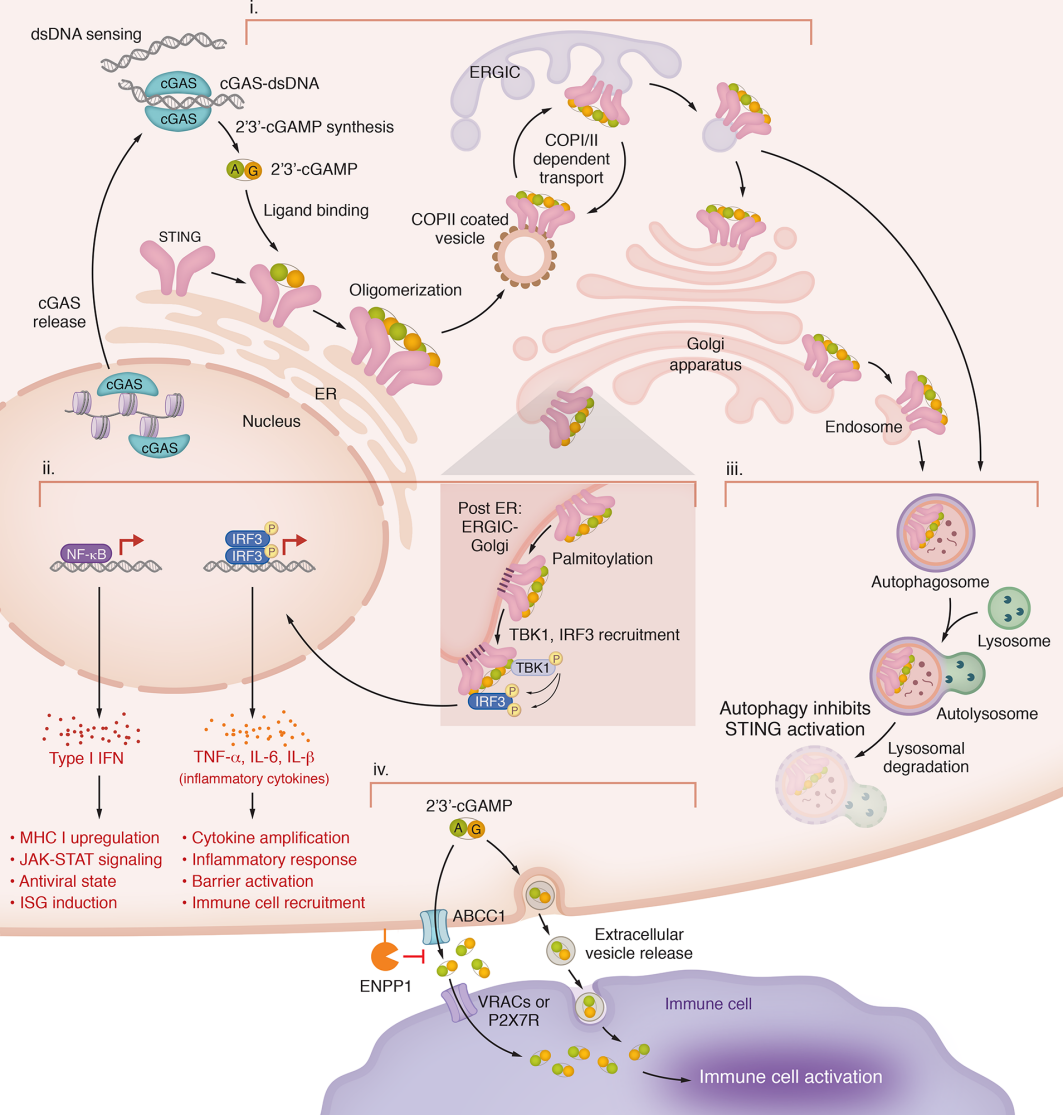
Spatiotemporal regulation and intercellular propagation of STING signaling. This schematic delineates the intracellular life cycle of STING signaling and its expansion into a paracrine communication network. (i) ER-to-Golgi activation axis. Upon binding to 2′3′-cGAMP, ER-localized STING dimers undergo conformational changes and oligomerization. This active complex exits the ER via COPII-coated vesicles, trafficking through the ER-Golgi intermediate compartment (ERGIC) to the Golgi apparatus. (ii) Signaling bifurcation at the Golgi. At the Golgi, STING undergoes palmitoylation, establishing a platform for divergent downstream outputs. The pathway bifurcates into the canonical TBK1/IRF3 axis (inducing type I IFN) and additional STING outputs, including NF-κB activation and LC3-associated autophagy-dependent clearance of cytosolic DNA. (iii) Signal resolution. To ensure transient immunity and restore homeostasis, the kinase ULK1 phosphorylates STING and attenuates IRF3-dependent signaling, while activated STING is subsequently routed to lysosomal degradation. Recent studies indicate that degradation of activated STING proceeds primarily via ESCRT-dependent lysosomal microautophagy rather than canonical macroautophagy. (iv) Intercellular cGAMP networking. Parallel to cell-intrinsic signaling, 2′3′-cGAMP functions as an immunotransmitter. Its extracellular availability is tightly governed by the balance between export pathways or channels (e.g., ABCC1, LRRC8-containing VRACs), hydrolytic degradation by ectonucleotidases (ENPP1, SMPDL3A), and uptake by bystander cells via specific transporters (e.g., SLC46A2, SLC19A1), thereby propagating paracrine innate immunity.

STING is an ER-resident adaptor protein that remains inactive under basal conditions to prevent autoimmunity (7). *TMEM173* (encoding STING) undergoes alternative splicing, generating distinct STING isoforms with differential signaling capacities, some of which attenuate canonical STING activation (92). The signaling cascade begins when 2′3′-cGAMP binds to the STING dimer, inducing a conformational rotation of the ligand-binding domain relative to the transmembrane domain (Figure 3A) (93, 94). This structural change leads to the formation of higher-order oligomers and exposes the C-terminal tail for downstream kinase recruitment (95, 96). Crucially, activation is coupled with intracellular trafficking. Upon ligand binding, STING exits the ER via COPII-coated vesicles and traffics through the ER-Golgi intermediate compartment to the Golgi apparatus (7, 97). At the Golgi, STING undergoes palmitoylation at conserved cysteine residues (C88/91 in human), a modification essential for creating lipid microdomains that serve as signaling platforms (98, 99). Beyond palmitoylation, STING is further regulated by additional PTMs, including ubiquitination. K27-linked polyubiquitination promotes STING activation by facilitating TBK1 recruitment, whereas K48-linked ubiquitination targets STING for degradation; conversely, removal of K48-linked ubiquitin chains by the USP18/USP20 axis stabilizes STING and sustains downstream signaling (100, 101).

Once assembled at the Golgi, STING functions as a bifurcated signaling hub (Figure 3B). The canonical inflammatory pathway is initiated when STING recruits TBK1 (102). TBK1 phosphorylates the STING C-terminal tail, which then recruits IRF3 for phosphorylation (102). Activated IRF3 dimerizes and translocates to the nucleus to drive type I IFN transcription, orchestrating antiviral and antitumor immunity (13). In addition, STING directs noncanonical functions independent of IFN. Through direct interaction with LC3 (ATG8) lipidation machinery, STING induces autophagy to clear cytosolic DNA and damaged organelles (13). Additionally, STING activates NF-κB and metabolic regulators, influencing cellular senescence and glycolysis (24, 103–105). The balance between these outputs, acute IFN versus autophagy or chronic NF-κB, depends on the signal strength and duration.

To prevent pathological hyperactivation, STING signaling must be precisely terminated. This resolution phase is governed by the autophagy/lysosome pathway. Following signal transmission, the kinase ULK1 phosphorylates STING (e.g., at S366), suppressing its activity and marking it for sorting into autophagosomes (Figure 3C) (106, 107). This ULK1-dependent degradation ensures that pathway activity is transient and restores cellular homeostasis. Although STING activation can promote LC3-dependent autophagy through direct interaction with the autophagy machinery (13), recent evidence indicates that degradation of activated STING itself occurs predominantly via lysosomal and endosomal microautophagy rather than canonical macroautophagy (108–111).

Disruptions at specific nodes of the “initiation-propagation-resolution” cycle act as primary drivers for distinct pathologies. Defects in the initiation and trafficking machinery are most commonly associated with monogenic autoinflammatory diseases. For instance, gain-of-function mutations in *TMEM173* that lower the threshold for oligomerization or disrupt the autoinhibitory state cause SAVI (23, 112). These mutations lead to constitutive TBK1 recruitment and chronic IFN signaling even in the absence of cGAMP ligand (99, 112). Similarly, mutations in the COPI complex subunit *COPA* impair the retrograde transport of STING from the Golgi back to the ER (113). In COPA syndrome, this trafficking defect results in the accumulation of activated STING at the Golgi, driving a pathogenic type I IFN signature that manifests as interstitial lung disease and arthritis (113, 114). Additionally, common human *TMEM173* alleles can act as genetic modifiers of STING-driven disease, as the HAQ STING haplotype was shown to dominantly dampen COPA-dependent STING signaling and associate with complete clinical nonpenetrance in COPA mutation carriers (115).

Conversely, sustained activation of the pathway driven by persistent cytosolic dsDNA is heavily implicated in age-related inflammatory, neurodegenerative, and autoimmune conditions. In homeostatic states, mitochondrial quality control and chromatin surveillance mechanisms limit the accumulation of mitochondrial or nuclear-derived dsDNA in the cytosol (21, 103, 116). However, mitochondrial dysfunction or genomic instability can result in chronic cytosolic dsDNA persistence, maintaining cGAS/STING activation and promoting inflammatory programs associated with senescence, neuroinflammation, and aging-related pathology (103, 116, 117). Impaired termination of STING signaling has also been implicated in neurodegenerative disease, where defective regulation of STING activity contributes to sustained inflammatory responses in the central nervous system (118). Furthermore, defects in the clearance of activated STING itself, normally mediated by ULK1-dependent autophagy, can perpetuate signaling, contributing to the pathogenesis of systemic lupus erythematosus (119, 120).

Finally, in the context of cancer, the pathway often undergoes a “propagation shift.” While acute cGAS/STING activation is essential for priming antitumor T cells, chronic low-level activation in established tumors can rewire downstream signaling (22). In chromosomal instability-high (CIN-high) tumors, sustained cGAS/STING signaling preferentially activates noncanonical NF-κB and STAT3 pathways while suppressing IFN responses (22, 121). This dysregulated output supports tumor metastasis, angiogenesis, and immune suppression, effectively converting a tumor-suppressive mechanism into a tumor-promoting one (41, 121).

## Intercellular 2′3′-cGAMP signaling, death pathways, and immune programming

Recently, the cGAS/STING pathway has been redefined not merely as a cell-intrinsic sensor, but as a mechanism for intercellular communication that transmits damage signals to the host immune system (9, 122). The second messenger 2′3′-cGAMP functions as a potent immunotransmitter that can be transferred to neighboring cells via gap junctions or viral particles to trigger STING activation in *trans* (Figure 3D) (8, 123–125). This “bystander activation” acts as a double-edged sword. In health, it alerts the immune system to localized infection or damage. However, in autoimmune conditions like systemic lupus erythematosus, excessive cGAMP transfer via gap junctions contributes to the pathological spread of inflammation to healthy tissues, exacerbating systemic damage (123).

In the context of cancer, this spreading effect is pivotal for the immunostimulatory effects of radiotherapy. Ionizing radiation induces double-strand breaks in tumor cells, generating high levels of 2′3′-cGAMP (27). Even if tumor cells themselves have silenced STING to evade detection, the cGAMP they generate can be released and taken up by infiltrating host immune cells, driving type I IFN production and antitumor immunity (27, 126). Thus, the intercellular transfer of cGAMP dictates the balance between effective tumor clearance and pathological inflammation.

The movement of 2′3′-cGAMP is actively regulated to maintain tissue homeostasis. The ABC transporter ABCC1 actively pumps cGAMP out of cells, limiting cell-intrinsic inflammation while facilitating paracrine signaling (127). Additionally, volume-regulated anion channels (e.g., LRRC8) have recently been identified as key conduits for cGAMP release (128, 129). Uptake is cell type specific. SLC19A1 (130) and SLC46A2 (131) function in myeloid cells to transport extracellular cGAMP. Beyond cGAMP spreading, tumor-derived extracellular vesicles have been shown to carry genomic or mitochondrial dsDNA to recipient cells (132). This DNA cargo allows antigen-presenting cells to sense tumor-derived dsDNA directly via their own cGAS, initiating an adaptive immune response even if the donor cancer cell lacks functional immune signaling (132, 133).

To prevent autoimmunity, the spread of cGAMP is constrained by extracellular hydrolysis. ENPP1 hydrolyzes extracellular cGAMP; downstream nucleotide metabolism can generate immunosuppressive adenosine (134, 161). While essential for preventing chronic inflammation in healthy tissues, ENPP1 is often hijacked by tumors (e.g., breast and lung cancer) to evade immune surveillance (134, 135). Similarly, macrophages induce SMPDL3A upon lipid metabolic stress to degrade cGAMP, preventing excessive inflammation in metabolic tissues (135). Therefore, the balance between cGAMP export (ABCC1) and degradation (ENPP1/SMPDL3A) distinguishes healthy immune surveillance from disease states.

The mode of cell death dictates the immunogenicity of the response. Immunologically silent apoptosis sequesters DNA, whereas lytic cell death (e.g., pyroptosis, necroptosis) releases dsDNA and cGAMP, amplifying inflammation (9, 122). Recent studies further demonstrate that STING activation can directly induce necroptosis, linking innate immune sensing to regulated inflammatory cell death (79, 136). In human myeloid cells, STING can trigger a lysosomal cell death program upstream of the NLRP3 inflammasome, linking DNA sensing to IL-1β maturation (137). Under severe stress, this can evolve into PANoptosis (coordinated by ZBP1), a massive cell death pathway that maximizes immune recruitment but risks tissue damage (138, 139).

Collectively, these findings frame the cGAS/STING axis as a dynamic intercellular network. The ultimate outcome — whether healthy homeostasis, autoimmune pathology, or antitumor immunity — depends on the efficiency of intercellular transfer and the stability of the immunotransmitter within the tissue microenvironment.

## Pathological rewiring of cGAS/STING signaling and therapeutic opportunities

**Figure 4:**
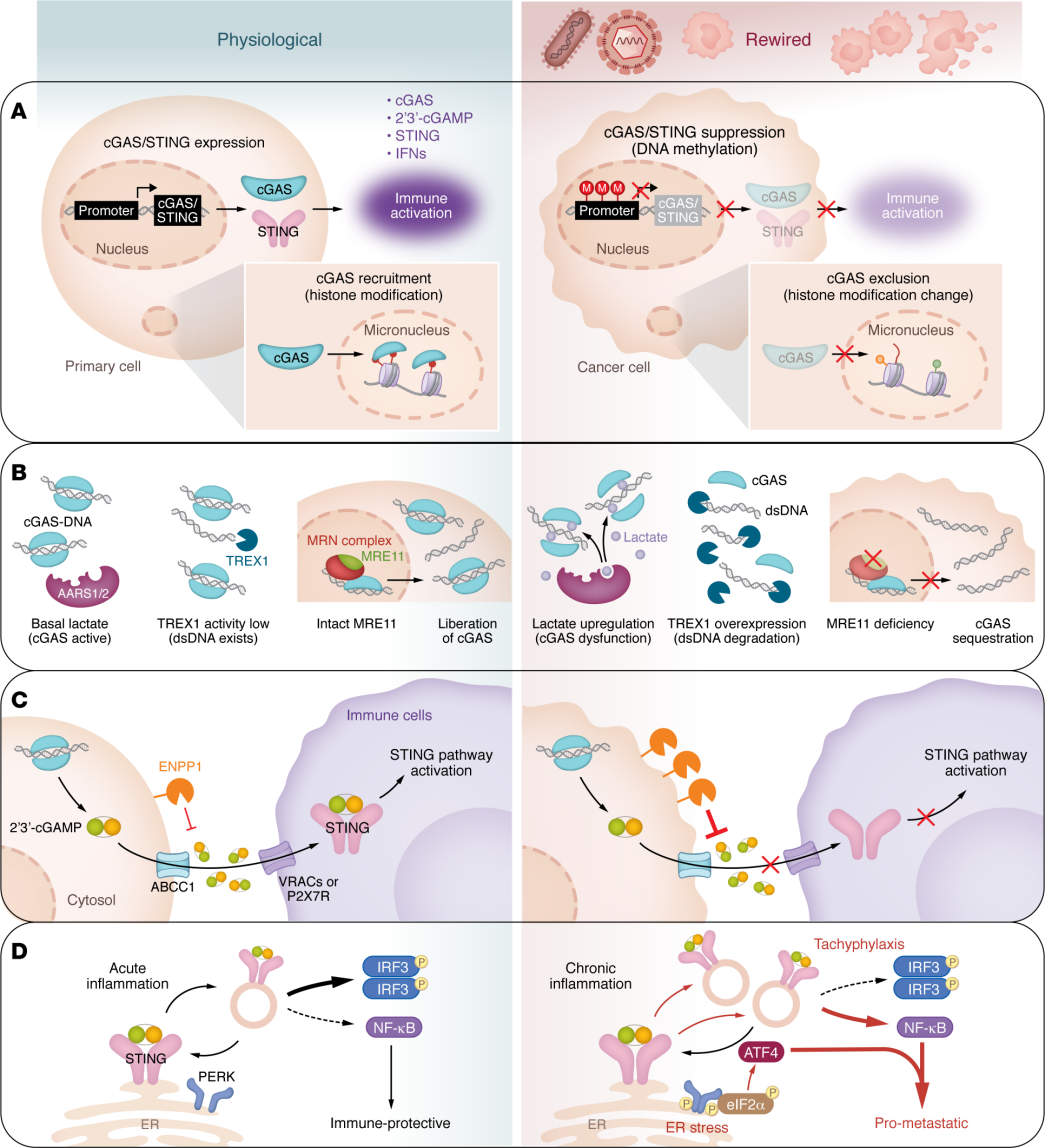
Multilevel rewiring mechanisms of the cGAS/STING pathway in pathological conditions. (**A**) Epigenetic and spatial regulation. In physiological states, cGAS and STING promoters are active, and specific histone modifications may facilitate cGAS recruitment to micronuclei or chromatin, thereby promoting immune activation. Conversely, cancer cells rewire this pathway via promoter hypermethylation (silencing expression) or altered histone marks that lead to cGAS exclusion and immune evasion. (**B**) Metabolic and enzymatic control of cytosolic DNA sensing. Under normal conditions, low TREX1 activity and intact MRE11 complexes facilitate the liberation and sensing of dsDNA by cGAS. In rewired states, the pathway is inhibited by the upregulation of lactate (which inhibits cGAS DNA sensing and activation through cGAS lactylation), overexpression of the exonuclease TREX1 (which degrades cytosolic dsDNA), or MRE11 deficiency (leading to cGAS sequestration). (**C**) Regulation of intercellular cGAMP transmission. Physiologically, 2′3′-cGAMP is exported via transporters such as ABCC1 and transferred to bystander immune cells through gap junctions or channels (VRACs, P2X7R) to trigger immune activation. In the rewired context, overexpression of ENPP1 degrades extracellular cGAMP, preventing immune cell activation. (**D**) Modulation of downstream signaling outcomes. While acute inflammation triggers primarily IRF3-dependent canonical immune-protective signaling, along with NF-κB activation, chronic STING activation–associated ER stress promotes tachyphylaxis. This rewires the pathway toward a noncanonical, prometastatic axis mediated by PERK-eIF2α-ATF4 signaling, associated with ATF4-dependent and NF-κB–related inflammatory programs rather than IRF3-centered signaling.

In human disease, cGAS/STING dysregulation most often reflects node-specific failure or rewiring rather than simple pathway loss. This is especially evident in cancer, IFN-driven inflammatory syndromes, and tissue injury states, where the same pathway can mediate either protective immunity or chronic pathology depending on how initiation, propagation, and termination are altered. This rewiring creates a paradoxical state where the pathway is present but functionally corrupted, serving as a driver of disease rather than a guardian of health. Similar to viral evasion strategies where pathogens encode specific proteins to sequester or cleave STING, tumors and diseased tissues exploit endogenous regulatory nodes to reshape immune outcomes. As illustrated in Figure 4, this remodeling occurs across four distinct layers: epigenetic licensing, metabolic gating, extracellular propagation, and transcriptional regulation.

The first level of rewiring involves the epigenetic regulation and physical accessibility of DNA to cGAS. As described above, cGAS expression and localization are tightly controlled. In physiological states, specific histone modifications (77, 78) may facilitate the recruitment of cGAS to chromatin or micronuclei to initiate surveillance. However, cancer cells often rewire this step via promoter hypermethylation to silence cGAS or *TMEM173* expression or by altering histone marks to exclude cGAS from chromatin, thereby preventing immune recognition (Figure 4A). Indeed, epigenetic silencing of *TMEM173* through DNA methylation has been reported across multiple tumor types, and pharmacologic inhibition of DNMT1 or EZH2 can restore STING pathway activity and enhance antitumor immunity (29–31).

Structurally, cGAS is normally tethered to the acidic patch of histones H2A-H2B, a “brake” mechanism that prevents autoreactivity. Recent studies highlight that this licensing is dynamic; the MRE11 complex is required to displace cGAS from nucleosomes in response to DNA damage, thereby allowing sensing to occur (79). In tumors lacking MRE11, cGAS remains sequestered and inactive despite high genomic instability (Figure 4B). Conversely, nuclear cGAS can be rewired to actively promote tumorigenesis; by interacting with PARP1 to disrupt the PARP1-Timeless complex, cGAS inhibits homologous recombination DNA repair and exacerbates genomic instability (140).

Superimposed on this structural regulation is metabolic and enzymatic rewiring. While low activity of the exonuclease TREX1 normally permits cGAS sensing, its overexpression in cancer degrades cytosolic dsDNA, removing the ligand for cGAS (19, 20, 74, 133, 141–143). Conversely, TREX1 loss or knockout can increase cytosolic DNA and reactivate cGAS/STING signaling, enhancing downstream IFN and inflammatory programs (144). Furthermore, the metabolic state of the tissue directly dictates immune signaling competence. For instance, in the setting of accumulating lactate in the glycolytic tumor microenvironment, the metabolic sensors AARS1 and AARS2 catalyze lysine lactylation of cGAS, impairing its ability to bind DNA and form active condensates (87). Additionally, efficient initiation requires cGAS to undergo liquid–liquid phase separation with DNA, a process drastically enhanced by cytosolic Mn²^+^ released from membrane-enclosed organelles (145). Alterations in intracellular ion homeostasis or phase separation factors can thus fundamentally alter the activation threshold.

Beyond intracellular initiation, the spatial propagation of the signal is heavily modulated. The second messenger 2′3′-cGAMP functions as an immunotransmitter, but its effective range is strictly controlled by the balance between transport and degradation. Overexpression of the ectonucleotidase ENPP1 is a common rewiring strategy in breast and lung cancers, allowing tumors to rapidly hydrolyze extracellular cGAMP and prevent the activation of antigen-presenting cells (Figure 4C) (130, 134). Concomitantly, differential expression of cGAMP transporters — such as the export channel ABCC1 and the SLC19A1 and SLC46A2 importers and LRRC8-containing volume-regulated anion channels (VRACs) — determines the directionality of the signal (129, 131, 146). A reduction in SLC46A2 expression in myeloid cells, for example, can sever the communication line between a dying tumor cell and the host immune system, effectively isolating the danger signal (131).

Finally, the duration of signaling can be rewired to alter the transcriptional output. While transient cGAS/STING activation drives an antiviral type I IFN response, chronic signaling, often sustained by CIN or defects in autophagy-mediated resolution, shifts the downstream trajectory. In this chronic state (tachyphylaxis), the pathway preferentially activates noncanonical NF-κB and PERK-dependent stress responses (Figure 4D) (121, 147). This output shift transforms STING from a tumor suppressor into a promoter of metastasis and tissue inflammation, driving disease progression in CIN-high cancers and autoinflammatory syndromes like SAVI (121, 147). Sustained STING signaling in this context can be pathologic, driving Treg and myeloid-derived suppressor cell differentiation while triggering apoptosis in T cells and DCs, shifting the balance from immune stimulation to immunosuppression (148, 149). These context-dependent constraints are increasingly informing how cGAS/STING-directed therapies are being designed and deployed.

## Translational considerations for cGAS/STING-directed therapy

These mechanistic insights suggest that the clinical relevance of the cGAS/STING pathway in cancer lies not only in whether the pathway can be activated, but also in how different therapies engage distinct regulatory nodes of the circuit (150–152). Radiotherapy, for example, can elicit antitumor type I IFN responses, yet recent work shows that this output may be blunted by inducible STING checkpoints in both tumor cells and host DCs, including HO-1–mediated disruption of cGAS/STING trafficking and YTHDF1-dependent lysosomal STING degradation (150, 151). In contrast, inactivation of CDK12/13 can increase immunostimulatory nucleic acids, drive STING-dependent T cell infiltration, and sensitize tumors to immune checkpoint blockade, highlighting how DNA damage response state may shape therapeutic responsiveness (152).

These observations suggest that direct pathway agonism alone may not be sufficient and that delivery context is likely to be especially important (153–157). First-in-human studies of intratumoral STING agonists support clinical feasibility, but recent preclinical work increasingly favors approaches that better control where and in which cells the pathway is engaged (153). Examples include restoration of cGAS expression in tumor cells to generate endogenous cGAMP that can activate surrounding immune cells, tumor cell–directed STING agonist antibody-drug conjugates, and tumor-targeted platforms that bias signaling toward IRF3/type I IFN programs (154–156). In parallel, ENPP1 inhibition can prolong extracellular cGAMP signaling and enhance responses to radiotherapy, chemotherapy, PD-1/PD-L1 blockade, or their combinations in preclinical models (157, 158).

Taken together, these findings support a more tailored translational framework in which pathway competence, suppressive checkpoints, signal propagation, and treatment timing are considered together rather than assuming that more STING activation is uniformly beneficial (150–158). Clinically, this suggests that cGAS/STING-directed therapy may be most effective when deployed as a context-specific adjunct to radiotherapy, chemotherapy, DNA damage response–targeted therapy, or immunotherapy and when coupled to delivery strategies that favor intratumoral or myeloid activation while limiting systemic inflammatory toxicity (152–158). This perspective also provides a rationale for viewing pathological rewiring not only as a mechanism of disease, but also as a guide for therapeutic intervention.

## Restoring immune homeostasis by targeting regulatory nodes

Our understanding of the cGAS/STING pathway has evolved far beyond its canonical definition as a cytosolic DNA sensor. As synthesized in this Review, the cGAS/STING pathway operates as a multifunctional regulatory node that integrates chromatin architecture, metabolism, and intercellular immune signaling to maintain tissue homeostasis (22, 122). While acute activation of this pathway is indispensable for antiviral defense and tumor immunosurveillance, we now understand that disease states, particularly cancer and chronic autoimmunity, rarely disrupt this circuit in totality. Instead, they exploit its multilevel regulation, leading to pathological rewiring that manifests as either epigenetic silencing, metabolic gating, or chronic noncanonical signaling (159, 160).

Understanding this multilevel rewiring provides the critical logic for next-generation therapeutic interventions. Early clinical efforts predominantly relied on first-generation STING agonists (e.g., DMXAA, cGAMP analogs) to bluntly “turn on” the pathway. The limited efficacy of these agents underscores the need for precision strategies that address specific regulatory defects rather than simple pathway activation (161).

First, for cold tumors, defined by poor signal propagation, the therapeutic bottleneck is often the failure of intercellular cGAMP transmission rather than cGAS generation itself. In these contexts, inhibiting the ectonucleotidase ENPP1 or enhancing the function of cGAMP transporters offers a mechanism to restore the paracrine delivery of immunotransmitters to antigen-presenting cells, thereby reigniting the cancer immunity cycle (162, 163).

Second, to overcome the toxicity and lymphopenia associated with systemic activation, the field is advancing toward targeted delivery. Systemic high-dose agonists often paradoxically induce apoptosis in T cells and DCs, depleting the very effectors required for immunity (164). Emerging antibody-drug conjugates that deliver STING agonists precisely to tumor cells or myeloid compartments represent a promising strategy to maximize intratumoral activation while sparing systemic immune populations (165).

Finally, in scenarios where the pathway is chronically hyperactivated and corrupted, such as in CIN-high metastasis or autoinflammatory syndromes like SAVI or neurodegenerative conditions linked to mitochondrial damage (e.g., Parkinson’s disease), further activation is detrimental. In these contexts, the pathway has shifted from an antitumor IFN response to a prometastatic NF-κB/PERK axis (121, 160). Here, the therapeutic goal is to reset the circuit. This could potentially be achieved using covalent STING inhibitors that block palmitoylation, autophagy enhancers that promote the lysosomal resolution of activated STING, or emerging PROTAC (proteolysis-targeting chimera) technologies that degrade the pathogenic STING protein entirely (13, 16).

In conclusion, the therapeutic targeting of cGAS/STING is shifting from a binary paradigm of activation versus inhibition to a more nuanced framework of regulatory tuning. By targeting specific nodes of rewiring, immune homeostasis may be more effectively reestablished to improve clinical outcomes in cancer and inflammatory diseases.

## Conflict of interest

The authors have declared that no conflict of interest exists.

## Funding support

This work is the result of NIH funding, in whole or in part, and is subject to the NIH Public Access Policy. Through acceptance of this federal funding, the NIH has been given a right to make the work publicly available in PubMed Central.

- NIH/National Cancer Institute grants (R37 CA227837, P50 CA058223, and R01 CA274254) to GPG.
- Department of Defense Breast Cancer Research Program (HT9425-23-1-0961 to GPG).
- V Foundation (T2019-010) to GPG.
- Breast Cancer Research Foundation to GPG.
- University of North Carolina Center for Triple Negative Breast Cancer to GPG.
- P01 CA247773 to GPG.
- T32 GM135128 to RL.

## Version 1 07/01/2026

Electronic publication
